# Dietary Intake Is Unlikely to Explain Symptom Severity and Syndrome-Specific Microbiome Alterations in a Cohort of Women with Fibromyalgia

**DOI:** 10.3390/ijerph19063254

**Published:** 2022-03-10

**Authors:** Amir Minerbi, Nicholas J. B. Brereton, Abraham Anjarkouchian, Audrey Moyen, Emmanuel Gonzalez, Mary-Ann Fitzcharles, Yoram Shir, Stéphanie Chevalier

**Affiliations:** 1 Institute for Pain Medicine, Rambam Health Campus, Haifa 3109601, Israel; 2 Ruth and Bruce Rapaport Faculty of Medicine, Technion-Israel Institute of Technology, Haifa 3525433, Israel; 3 Institut de Recherche en Biologie Végétale, University of Montreal, Montreal, QC H1X 2B2, Canada; nicholas.brereton@umontreal.ca; 4 School of Human Nutrition, McGill University, 21111 Lakeshore Rd, Montreal, QC H9X 3V9, Canada; abraham.anjarkouchian@mail.mcgill.ca (A.A.); audrey.moyen@mail.mcgill.ca (A.M.); stephanie.chevalier@mcgill.ca (S.C.); 5 Canadian Centre for Computational Genomics, McGill University and Genome Quebec Innovation Centre, 740 Docteur Penfield Ave, Montreal, QC H3A 0G1, Canada; emmanuel.gonzalez@mcgill.ca; 6 Microbiome Research Platform, McGill Interdisciplinary Initiative in Infection and Immunity, Genome Center, McGill University, 740 Docteur Penfield Ave, Montreal, QC H3A 0G1, Canada; 7 Alan Edwards Pain Management Unit, McGill University Health Centre, 1650 Cedar Ave, Montreal, QC H3G 1A4, Canada; mary-ann.fitzcharles@mcgill.ca (M.-A.F.); yoram.shir@muhc.mcgill.ca (Y.S.); 8 Division of Rheumatology, McGill University Health Centre, 1650 Cedar Ave, Montreal, QC H3G 1A4, Canada; 9 Research Institute of the McGill University Health Centre, 1001 Décarie Blvd, Montreal, QC H4A 3J1, Canada; 10 Department of Medicine, McGill University, 845 Sherbrooke St. W, Montreal, QC H3A 0G4, Canada

**Keywords:** fibromyalgia, pain, nutrition, microbiome

## Abstract

Background: Significant alterations were recently identified in the composition and putative function of the gut microbiome in women with fibromyalgia. As diet can influence the composition of the gut microbiome, differences in nutritional intake could, in theory, account for some of these specific fibromyalgia microbiome alterations. The current study aims to compare the diet of women with fibromyalgia to that of controls in order to explore possible associations between the intake of certain nutrients, symptom severity and gut microbiome composition. Methods: The study population was comprised of 56 women with fibromyalgia and 68 controls. Dietary intake was assessed using the NIH Automated Self-Administered 24 h recall, following dietitian’s instructions and the completion of a three-day dietary recall. The gut microbiome was assessed by 16S ribosomal RNA gene sequencing of stool samples. Results: Most demographic and anthropometric characteristics were comparable between groups. The average energy and macronutrient intake (total and relative) and overall diet quality score were not different between patients and controls, nor were the main vitamins, minerals, fatty acids, alcohol, caffeine, sugar or fiber intakes. The daily intake of micronutrients and normalized macronutrients in women with fibromyalgia was largely not correlated with disease-specific measures, including pain intensity, fatigue, cognitive symptoms and quality of sleep, or with the relative quantity of almost any of the gut microbiome bacterial taxa differentially abundant in fibromyalgia. Conclusion: These data demonstrate that dietary intakes, as evaluated by self-reported questionnaires, probably cannot explain the syndrome-specific differences in gut microbiome or the clinical phenotype of fibromyalgia.

## 1. Introduction

Fibromyalgia is a prevalent syndrome characterized by chronic widespread pain, fatigue, cognitive symptoms and disturbed sleep [1,2,3]. Affecting 2–4% of the adult population, fibromyalgia leads to a considerable decrease in the quality of life of affected individuals [1,4]. Currently, there is no known cure for fibromyalgia, and recommended treatment is multi-disciplinary, aiming to alleviate symptoms [1,5]. Accepted treatment modalities include lifestyle modification, pharmacotherapy, physical measures and psychosocial support [1,5,6]. Lifestyle measures are considered as of paramount importance and include graded physical activity, sleep hygiene, pacing of daily activity and dietary interventions. Different dietary regimens have been tested in individuals with fibromyalgia, with variable levels of success [7,8,9].

The pathophysiology of fibromyalgia is poorly understood, and few consistent objective findings have been demonstrated in patients [5]. Recently, we identified the significant alterations in the composition and function of the gut microbiome in individuals with fibromyalgia, which were quantitatively correlated with symptom severity [10]. Changes in the composition of the gut microbiome in individuals with fibromyalgia have also been recently reported by Clos-Garcia et al. [11]. These observed correlations, linking the composition and putative function of the gut microbiome to fibromyalgia, do not infer causality; to date, it is not possible to determine whether microbiome alterations play a role in the pathogenesis of fibromyalgia or are merely a result of the syndrome.

The composition of the gut microbiome is affected by multiple inherent and environmental factors, of which diet seems an important contributor [12,13]. Significant alterations in the composition of the gut microbiome are observed in as little as a few days following changes in dietary intake [13,14,15]. Furthermore, changes in the composition and function of the gut microbiome have been suggested to be involved in the beneficial effects of specific dietary interventions in certain medical conditions, including irritable bowel syndrome (IBS) [16,17,18] and epilepsy [19].

Several studies reported on the differences in the dietary habits of individuals with fibromyalgia [20,21,22]. The purpose of this study is to determine whether diet is associated with either previously defined fibromyalgia-specific alterations in the gut microbiome or the severity of symptoms in adult women with fibromyalgia.

## 2. Methods

### 2.1. Study Design and Oversight

The study was conducted at the Alan Edwards Pain Management Unit (AEPMU) of the McGill University Health Centre, Montreal (MUHC), and at the West Island Rheumatology Clinic, in Montreal, QC, Canada. The study was approved by the MUHC institutional review board (approval number 2018-3574). Participants were given a detailed explanation of the study and signed an informed consent form.

### 2.2. Patient Recruitment and Clinical Evaluation

Women with fibromyalgia were recruited either by advertisements in the local media, by a dedicated website or directly by their treating physician. The inclusion criteria for patients were: female sex, aged 30–60 years, widespread pain index (WPI) and symptom severity scores compatible with the American College of Rheumatology diagnostic criteria (ACR 2016) for fibromyalgia, and ability to give informed consent in French or English. Control participants were recruited by referral from patient participants and advertisements in the local media and a dedicated website. Control participants of three separate groups were recruited: (1) first-degree adult female relatives of patients participating in the study were recruited as genetic controls (RC); (2) adult household members of patients participating in the study were recruited as environment controls (HC); and (3) unrelated healthy adult women, 30–60 years of age (UC).

The exclusion criteria were: any acute illness, change in regularly taken medication or substantial dietary alterations in the preceding month, antibiotic treatment in the preceding two months or any major comorbid illness (e.g., malignancy, active inflammatory disease, and metabolic disease).

Patients with chronic pain were interviewed by a specialized pain physician for a thorough clinical assessment and only those whose diagnosis of fibromyalgia was confirmed were included in the study. All participants—patients and controls—were interviewed by the same physician, and data were collected regarding their demographics, anthropometrics, co-morbidities, medications, dietary intake, smoking and alcohol consumption. All participants completed the following questionnaires: the Fibromyalgia Survey Diagnostic Criteria and Severity Scale (FSDC) questionnaire, assessing symptom severity, pain location, fatigue, sleep quality and cognitive and somatic complaints in fibromyalgia patients [23]; the Fibromyalgia Impact Questionnaire (FIQ), a 10-item questionnaire evaluating physical functioning, work difficulty, pain, fatigue, morning tiredness, stiffness, anxiety and depression [24]; and the Insomnia Severity Index (ISI) Sleep Scale from the Medical Outcomes Study, a 12-item sleep quality evaluation questionnaire [25]. All questionnaires were available in English or in French and have been validated in both languages [26,27,28,29]. All participants were interviewed by a dietitian, with instructions for completing the web-based NIH ASA24-Canada dietary recall, validated in English and in French [30].

### 2.3. Dietary Intake Assessment

Dietary intake was assessed using the NIH Automated Self-Administered 24 h recall (ASA24-Canada version 2016, English or French) as previously described. All participants were trained and supervised by a dietitian as they completed the first day of recall. They were then instructed to complete the dietary assessment on 2 additional days during the following week, allowing for a total of 3 days of dietary assessment, of which 2 were weekdays and 1 was a weekend day. Datasets with less than two completed days were discarded. Daily nutrient averages were calculated, and macro- and micronutrient intakes (including supplements) were compared between groups as absolute intakes and relative to energy intake or body weight for macronutrients. Under-reporting was investigated by applying a ratio of reported energy intake over resting energy expenditure estimated with the Mifflin–St. Jeor equation [31]; participants with a ratio below 0.9 were deemed as under-reporters and excluded from the analysis. Finally, the Healthy Eating Index (HEI-2015) was calculated to assess diet quality [32].

### 2.4. Sample Acquisition and Handling

Stool samples for microbiome analysis were collected as previously reported [10]. Briefly, samples were collected at patients’ homes using the OMNIgene Gut OM-200 kit (DNA Genotek, Ottawa, ON, Canada), and fresh frozen at −20 °C. Samples were shipped on ice, in controlled shipping conditions, and were stored at −80 °C upon arrival at the research facility.

### 2.5. DNA Extraction and 16S Ribosomal rRNA Gene Amplification and Sequencing

DNA extraction, amplification and sequencing were performed as previously reported [10]. Briefly, DNA was extracted from all stool samples using the QIAamp PowerFecal DNA kit (Qiagen: Venlo, The Netherlands) and the V5-V6 region (based on *Escherichia coli*) of the 16S ribosomal RNA (rRNA) was targeted for amplification by PCR using the forward primer: S-D-Bact-0785-a-S-18, GGMTTAGATACCCBDGTA and reverse primer: S-*-Univ-1100-a-A-15, GGGTYKCGCTCGTTR [33]. The CS1 (ACACTGACGACATGGTTCTACA) and CS2 (TACGGTAGCAGAGACTTGGTCT) tags were used to add a barcode and Illumina adapters. Sequencing was performed using the MiSeq250 platform (2 × 250 nucleotides (nt) paired-end sequencing). The ANCHOR pipeline was used to process and annotate sequence reads [10,34] (https://github.com/gonzalezem/ANCHOR, accessed on 9 March 2022). Sequences were aligned and dereplicated using Mothur [35] before the selection of operational taxonomic units (OTUs) using a count threshold of 48 across all samples. Annotation used 4 sequence repositories with strict BLASTn criteria (>99% identity and coverage): NCBI curated bacterial and Archaea RefSeq, NCBI nr/nt, SILVA, Ribosomal Database Project (RDP) (NCBI curated bacterial and Archaea RefSeq was given a priority when at 100% identity and coverage). In the cases where the highest identity/coverage was shared amongst different putative annotations, all were retained and reported. Amplicons with low counts (<48) were binned to high-count sequences in a second BLASTn, using a lower threshold of >98% identity/coverage.

### 2.6. DESeq2 Differential Abundance Analysis

Differential abundance of OTUs in fibromyalgia versus unrelated controls was calculated using DESeq2 [36,37], as previously reported [10], with a selection parameter of false discovery rate (FDR; Benjamini–Hochberg procedure) <0.1 [38,39]. Regularized log transformation was applied to raw counts across samples (rlog function, R phyloseq package). Sparsity and low-count cut-offs were used whereby an OTU count in a single sample is <90% of the count in all samples, and the OTU count must be >2 in 40% of the samples.

### 2.7. Correlation between Nutritional Measures, Taxa Abundance and Clinical Indices

Correlations between the normalized nutrient intake, log-transformed OTU abundance, and clinical variables were calculated using the Kendall rank correlation. The individual *p*-value was calculated for each comparison, and the correction for multiple comparisons was performed using Benjamini–Hochberg FDR, with a cut-off value of 0.05. Results were visualized using color-coded correlation matrices representing the correlation coefficients, which were sorted based on hierarchical clustering using MathWorks MATLAB 2020a.

### 2.8. General Statistical Considerations

Demographics, anthropometrics, co-morbidities and medications were compared using ANOVA for univariate comparisons and MANOVA for multivariate comparisons, on IBM^®^ SPSS^®^ Statistics version 23 and MathWorks MATLAB^®^ version 2020a.

Dietary analysis was performed as follows: Kolmogorov–Smirnov and Shapiro–Wilk tests were used to test the normality of distributions. Dietary intake data were compared between all groups with the one-way ANOVA and post hoc Games-Howell test (appropriate for unequal sample size) unless otherwise stated. The intake of nutrients, which was not normally distributed, was analyzed using the non-parametric Kruskal–Wallis test. Analyses were conducted using IBM^®^ SPSS^®^ Statistics version 23.

## 3. Results

### 3.1. Participant Characteristics

A total of 200 patients were screened for this study, of which 29 were excluded during screening, and 15 were excluded following a medical interview that ruled out the diagnosis of fibromyalgia. Of the remaining 156 participants, 12 were excluded for incomplete filling of the dietary recall questionnaires and 20 were excluded for under-reporting (Supplementary Figure S1). Of the 124 participants who were included in the analyses, 56 were women with fibromyalgia, and 68 were healthy participants in 1 of the 3 control groups: 10 were first-degree relatives of fibromyalgia patients (RC), 18 were household members of fibromyalgia patients (HC) and the remaining 40 were unrelated controls (UCs). Other than sex and marital status, the differences in demographic and anthropometric characteristics among groups were not statistically significant (Table 1). All household members were male, while participants in all other groups were women. No seasonal variation in recruitment was observed between groups when comparing sample collection season (ANOVA *p* = 0.36, F = 1.08).

Fibromyalgia patients were women (mean age: 47 ± 8 years), who on average had been diagnosed with fibromyalgia 12 (SD 9.7) years earlier. Ninety-three percent of all participants were of Caucasian ethnicity. The mean WPI in the patient group was 10 (SD 3.3), and the mean symptom severity score (SSS) was 9.2 (SD 1.8). The American College of Rheumatology diagnostic criteria (ACR 2016) and FIQ scores were significantly higher in patients compared to all control groups (Pillai’s trace *p* < 0.0001, F = 3.86; Supplementary Table S1). IBS was significantly more prevalent among participants with fibromyalgia (48%, *p* < 0.0001, Supplementary Table S1).

### 3.2. Dietary Assessment

Dietary assessment was performed for all participants using the NIH Automated Self-Administered 24 h recall. The energy and macronutrient intake (total and relative) did not differ between patients and controls (Table 2). In addition, vitamin, mineral, different fatty acids, alcohol, caffeine, sugar and fiber intakes did not differ between groups (Table 3). The overall diet quality score was calculated for all eligible participants and no differences were observed between the groups (Table 3). Non-metric multidimensional scaling (NMDS) analysis of the daily food and nutrient intake showed an overlapping pattern of fibromyalgia patients and controls, whereby the difference between the groups was statistically insignificant (R^2^ = 0.001, *p* = 0.88), further corroborating the similarity of dietary intake in both groups (Supplementary Figure S2).

### 3.3. Nutritional Supplements

Nutritional supplements were taken more frequently by participants with fibromyalgia (70%) as compared to all control groups (RC 40%, HC 0%, UC 35%). However, this observation did not reach statistical significance (ANOVA *p* = 0.1), nor did the consumption of any individual supplement (Supplementary Table S2).

### 3.4. Dietary Intake Is Not Correlated with the Severity of Fibromyalgia Symptoms

**Figure 1:**
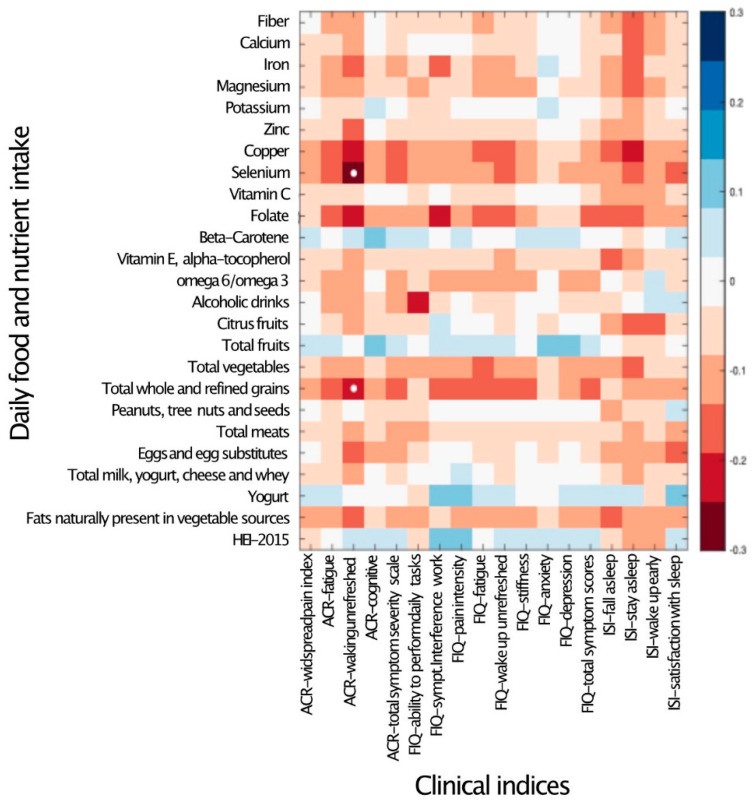
Daily nutrient and food intake is mostly not associated with clinical indices. Heat map of a univariate Kendall correlation matrix between the daily intake of fiber, selected micronutrients and food groups (*y*-axis) vs. clinical covariates (*x*-axis): disease severity metrics (FMDC: 2016 fibromyalgia diagnostic criteria); quality of life scores (FIQ); and sleep quality (sleep) scores. Blue shades indicate positive correlations while red shades indicate negative correlations (−0.5 < tau < 0.5). Statistically significant correlations are marked by a white circle (Benjamini–Hochberg FDR < 0.05). Food intake units are provided in Table 3. ACR–American College of Rheumatology Criteria; ISI–Insomnia Severity Index; and HEI 2015–Healthy Eating Index 2015.

To explore the possible quantitative correlation between participants’ dietary intake and symptom severity, the daily intake of micro- and macronutrients was correlated with the clinical indices of fibromyalgia. Other than waking up unrefreshed, which was negatively correlated with the intake of selenium and grains, no significant correlations were detected between the intake of any nutrient and the severity of any symptoms (Figure 1).

### 3.5. Overall Gut Microbiome Composition Is Correlated with Dietary Intake

**Figure 2:**
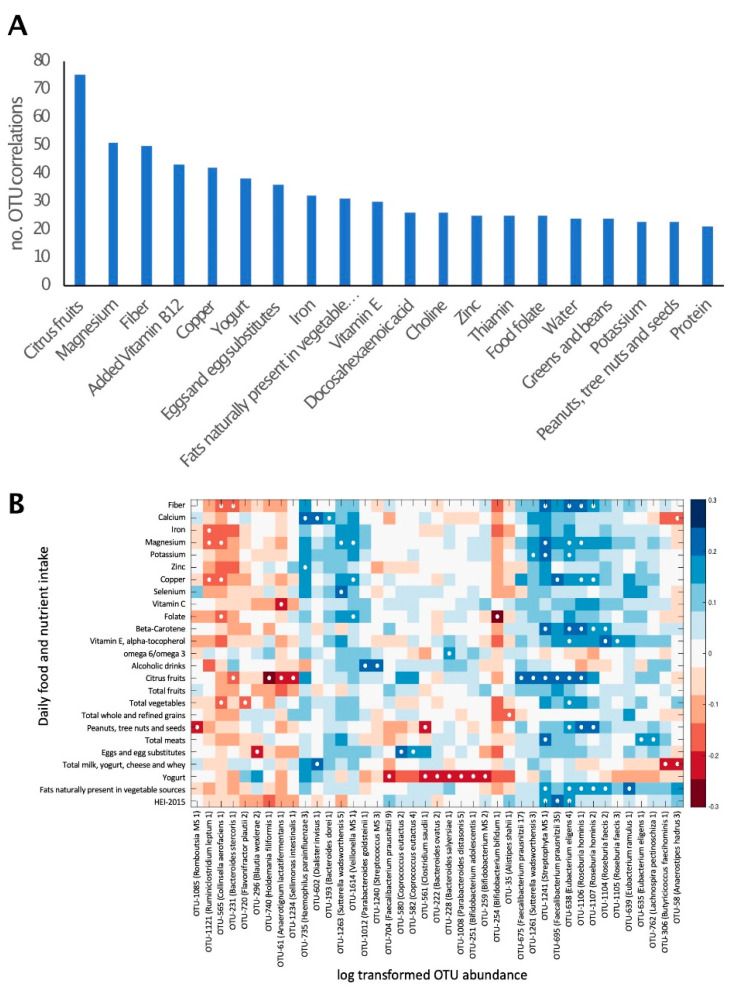
Daily nutrient and food group intake is correlated with gut microbiome composition. (**A**). The number of bacterial taxa showing significant (*p* < 0.01) correlation with the intake of selected nutrients and food groups. Twenty dietary factors showing correlations to the highest number of bacterial taxa are presented. (**B**). Heat map of a univariate Kendall correlation matrix of selected taxa abundance (log2; *x*-axis) vs. daily food and nutrient intake (*y*-axis). Heatmap is sorted based on a hierarchical clustering of OTUs. Blue shades indicate positive correlations while red shades indicate negative correlations (−0.3 < tau < 0.3). Statistically significant correlations are marked with a white circle. Food intake units are provided in Table 3. HEI 2015–Healthy Eating Index 2015.

The correlation of macro- and micro-nutrient intake with the relative abundance of all bacterial taxa was explored. A total of 1831 significant correlations were observed (*p* < 0.01), corresponding to 574 OTUs. These comprised 129 OTUs, which could be identified as putative species. The number of bacterial taxa for which a significant correlation with the intake of certain nutrients was observed differed between nutrients. This number ranged from only 1 correlated taxon for poultry consumption to 75 correlated taxa for citrus fruits (Figure 2A). The nutrients or foods that correlated with the highest number of bacterial taxa were citrus fruits, magnesium, fiber, copper and yogurt.

Well-characterized members of the phyla firmicutes, including *Roseburia hominis*, *Eubacterium eligens* and *Eubacterium ramulus*, were positively correlated with the consumption of nuts, vegetables and vegetable oils, respectively. The actinobacteria *Bifidobacterium bifidum* was negatively correlated with folic acid (Figure 2B). Yogurt consumption was negatively correlated with the abundance of several bacterial species, including *Bifidobacterium adolescentis*.

### 3.6. Dietary Intake Is Not Associated with Most Fibromyalgia-Specific Microbiome Alterations

Seventy-two bacterial taxa were found at either significantly higher or lower relative abundance in fibromyalgia patients as compared with controls (as previously reported [10]). To test the possibility that some of these differences could be attributable to altered dietary intake, the relative abundance of the differentially abundant (DA) bacterial taxa was correlated with the daily intake of all measured micro- and macronutrients. No significant correlations were observed between any measure of dietary intake and the relative abundance of DA bacterial taxa.

**Figure 3:**
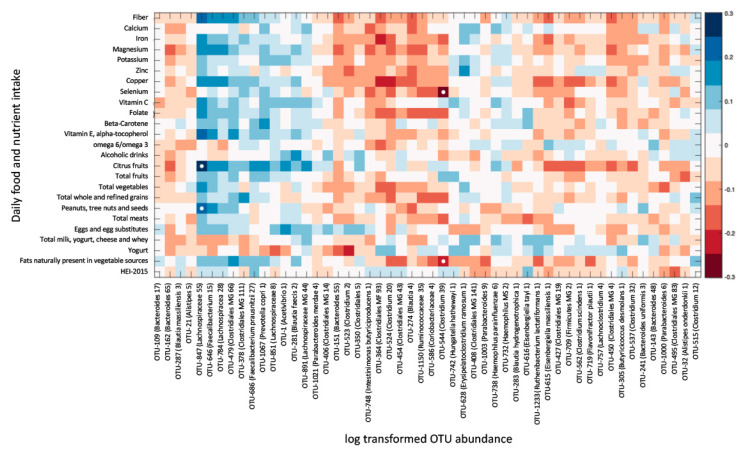
Daily nutrient intake is mostly not associated with the relative abundance of OTUs differentially abundant (DA) in fibromyalgia. Heat map of a univariate Kendall correlation matrix between DA taxa abundance (log2; *x*-axis) and selected daily macro- and micronutrient, and food group intake (*y*-axis). Heatmap is sorted based on a hierarchical clustering of DA OTUs. Blue shades indicate positive correlations while red shades indicate negative correlations (−0.3 < tau < 0.3). Statistically significant correlations (BH FDR *p* < 0.05) are marked with a white circle. Food intake units are provided in Table 3. HEI 2015–Healthy Eating Index 2015.

To verify that significant correlations were not lost due to correction for multiple comparisons, the correlation was explored between DA taxa abundance and the intake of a subset of nutrients, which were found to have the highest numbers of correlated species. Significant correlations were only observed between 2 OTUs (identified to the genus level) and the intake of citrus fruits, vegetables, nuts and selenium (Figure 3). In conclusion, of the 72 taxa differentially abundant in fibromyalgia, only 2 had potential correlations with dietary factors, and of the 19 species/strains differentially abundant in fibromyalgia, none correlated with dietary intake.

## 4. Discussion

In this cohort of women with fibromyalgia and healthy controls, dietary intake did not correlate with symptom severity and syndrome-specific microbiome alterations. In the present study, clinical measures, nutritional intake and microbiome composition were analyzed for a cohort of women with fibromyalgia and control groups comprising household members, first-degree relatives and unrelated healthy women. Women with fibromyalgia did not differ in their demographic and anthropometric measures when compared to unrelated and first-degree control group participants. When comparing their nutritional intake, average energy intake, macronutrient intake (total and relative) and overall diet quality score, no differences were observed between patients and controls. Furthermore, vitamin, mineral, fatty acids, alcohol, caffeine, sugar and fiber intakes were similar between the groups. The normalized daily intake of macro- and micronutrient intake did not correlate with the severity of fibromyalgia symptoms, including pain intensity, fatigue, cognitive symptoms and sleep problems.

At first look, these results may seem to contradict previous reports on dietary habits among individuals with fibromyalgia. Indeed, two surveys of nutritional habits of fibromyalgia patients reported a high consumption of nutritional supplements and frequent adherence to certain diets. Arranz et al. reported that 30% of fibromyalgia patients had changed their diet following the diagnosis, and 74% of them consumed nutritional supplements [20]. López-Rodríguez et al. similarly reported that 47% of fibromyalgia patients (but also 58% of healthy controls) adhered to restrictive diets while 86% used nutritional supplements [22]. Similar to these two previous studies, 70% of fibromyalgia patients in the present study reported nutritional supplement use, but this use did not differ significantly from the control groups. Overall dietary habits and diet quality assessed with the HEI-2015 tool [32] were also similar for patients and controls. A recent study using the slightly different HEI-2010 tool reported better self-efficacy and less depression associated with dietary quality in 36 women with fibromyalgia [40]; relationships not found in our study. Diet quality in that study was assessed from a non-quantitative food frequency questionnaire, likely to overestimate food intake [41]. The overall diet quality score of 57 (out of 100) calculated in our study was indeed lower, and typical of that reported for the U.S. adults in the National Health and Nutrition Examination Survey 2011–12 (*n* = 7935) [42], that is 56.6, with a similar distribution range (from 28 to 92) to that reported in a large U.S. multi-ethnic cohort (*n* > 215,000) [43], adequately wide for correlation analysis.

In consideration of the composition of the gut microbiome, the abundance of certain taxa significantly correlated with dietary intake. These included several associations that were biologically coherent. For example, *Roseburia* sp. are often identified as associated with dietary factors in microbiome studies [44,45]. Here, the association of *Roseburia hominis* with the dietary intake of nuts and carotene, a marker of vegetables and fruit consumption (Figure 2B), although not previously observed, is consistent with the species identified capability for β-mannan degradation (the backbone of softwood hemicellulose, but also predominant in the endosperm of many nuts and seeds) [46]. Correlations of the gut microbiome composition with the intake of insoluble fibers were also observed here: plant cell walls (insoluble fiber) are one of the most impactful factors on the gut microbiome community as they are dense in complex carbohydrates. Humans do not produce cellulase enzymes capable of cell wall deconstruction and thus rely on bacterial degradation. *Eubacterium eligens* was positively associated with a number of vegetable and fiber dietary factors and is very well characterized as a pectin (plant primary cell wall) degrader [47], while *E. ramulus*, a flavonoid-degrading bacteria (a class of plant secondary metabolites [48]), was positively correlated with diets rich in plant-derived fats [49]. Other associations could also be observed, such as the significant inverse correlation of *Bifidobacterium bifidum* with folic acid, which could also be expected as the species is one of the few major folic acid producers within the human gut microbiome [50], and increased folic acid intake should, in theory, reduce the value of a high production niche. Alterations in certain *Bifidobacterium* species were observed in yogurt consumers. Thus, the dietary intake of certain foods seems intimately correlated with the relative abundance of multiple bacterial species in the gut.

Alterations in the gut microbiome have been described in several conditions of chronic pain, including fibromyalgia [10,11], and its role in the pathogenesis of chronic pain has been suggested in animal models [51]. To date, the association between chronic pain and changes in the composition of the gut microbiome in humans is correlative rather than causal [10]. Our findings indicate that dietary alterations do not explain the *specific* microbiome alterations observed in fibromyalgia patients; the abundance of most bacterial taxa, which were differentially abundant in fibromyalgia, did not correlate with the intake of any micro- or macronutrient.

Diet is considered one of the key variables shaping the composition of the gut microbiome [14,15]. Dietary interventions have been suggested as a potential treatment modality for fibromyalgia and chronic pain [9,52,53,54], and individuals with fibromyalgia often explore nutritional adjustments in an attempt to control their symptoms [20]. The notion that dietary alterations may have a role in symptomatic control is further corroborated by reports that the introduction or elimination of certain foods can have an effect on certain symptoms, such as fatigue, pain and gastrointestinal discomfort [55]. It is thus somewhat surprising that the dietary intake of individuals with fibromyalgia was not different from a cohort of controls (both related and unrelated to patients), and that specific alterations in the vast majority of bacterial species observed in fibromyalgia patients were not correlated with the intake of any measured macro- or micro-nutrient. These findings suggest that both the symptoms of fibromyalgia, and the gut microbiome alterations associated with it, are probably not maintained by nutrition differences but rather by other factors, either environmental, intrinsic or both. The observed significant correlations between certain bacterial taxa and nutrient intake (Figure 2) demonstrate that the study was sufficiently powered to detect such correlations, suggesting that the lack of correlation between dietary intake, symptom severity and fibromyalgia-related DA bacterial taxa quantities (shown in Figure 1 and Figure 3) cannot be attributed to an underpowered cohort.

This study has several limitations. First, participants were generally a homogeneous cohort in terms of ethnicity and geographical distribution. Second, the dietary recall, despite being a well-validated tool, may lead to inaccurate reporting, specifically due to recall and reporting biases. Objective measures of nutrient intake, when applicable, would be required to strengthen these observations. This research did not explore fibromyalgia-related microbiome interactions with dietary interventions or specific diets, such as Mediterranean or vegetarian, and this traditional diet assessment tool does not evaluate non-nutrient component, such as polyphenols, or that could potentially affect the microbiome [56,57]. Lastly, the collection and analysis of a large dataset, which necessitates statistical correction for multiple comparisons, could potentially fail to detect weak correlations.

## 5. Conclusions

Our data suggest that dietary intake is unlikely to explain the differences in gut microbiome or the clinical phenotype of fibromyalgia. Contrary to the belief that fibromyalgia patients commonly adhere to dietary adjustments, fibromyalgia patients and controls consumed similar diets. A better understanding of the intricate interactions between chronic pain, diet and the microbiome calls for further studies, prospectively exploring the effects of dietary interventions on clinical symptoms as well as on the composition of the gut microbiome in individuals with fibromyalgia.

### Significance and Innovation

Despite the common belief that diet could impact pain and other symptoms of fibromyalgia, in this cohort, dietary intake did not correlate with symptom severity.

The dietary intake of fibromyalgia patients was not different from that of the matched controls.

While the intake of macro- and micronutrients was strongly correlated with the overall composition of the gut microbiome, it was not associated with the abundance of specific bacterial taxa known to be altered in fibromyalgia.

## Figures and Tables

**Table 1:** Selected demographic and anthropometric characteristics of fibromyalgia patients and controls.

	FM (56)	First-Degree Relatives (10)	Household Members (18)	Unrelated Controls (40)	ANOVA *p*
Sex	Women	Women	Men *	Women	<0.0001
Age (years)	47 ± 8	44 ± 17	47 ± 10	44 ± 9	0.58
Married (%)	54%	40%	94% *	58%	0.01
No. of children	1.5 ± 1.2	1.1 ± 1.45	1.0 ± 1.1	1.4 ± 1.3	0.38
No. of household members	2.6 ± 1.4	3.2 ± 1.6	2.7 ± 1.1	3.0 ± 1.6	0.74
Academic education	77%	70%	72%	88%	0.42
Ethnicity, maternal (%caucasian)	96%	100%	100%	90%	0.27
Ethnicity, paternal (%caucasian)	93%	100%	100%	88%	0.29
Occupational status (% working)	64%	60%	83%	73%	0.41
Smoking	9%	0%	6%	8%	0.74
BMI	29.6 ± 7.4	28.3 ± 7.1	28.7 ± 5.4	28.5 ± 7.3	0.36

Mean ± SD or % as indicated. * indicates significant difference (*p* < 0.01) between household members and all other groups.

**Table 2:** Daily average intakes of macronutrients.

	FM (56)	First-Degree Relatives (10)	Household Members (18)	Unrelated Controls (40)
Energy (kcal)	1940 ± 460	1936 ± 322	2180 ± 552	2008 ± 600
Energy (kcal/kg)	28 ± 11	24 ± 5 *	34 ± 11	31 ± 12
Protein (g)	76 ± 25	83 ± 24	89 ± 31	81 ± 24
Protein (g/kg)	1.1 ± 0.5	1.1 ± 0.4	1.4 ± 0.6	1.2 ± 0.4
Protein (% of E)	16 ± 4	17 ± 4	16 ± 4	15 ± 3
Carbohydrates (% of E)	47 ± 10	48 ± 6	46 ± 5	49 ± 9
Sugars (g) ^1^	108 ± 45	91 ± 36	98 ± 36	110 ± 62
Fibers (g) ^2^	19 ± 7	16 ± 5	18 ± 5	23 ± 12
Lipids (% of E)	36 ± 7	35 ± 5	37 ± 5	35 ± 7
Saturated (% of E)	11 ± 4	12 ± 2	12 ± 3	11± 4
Monounsaturated (% of E)	14 ± 4	13 ± 2	14 ± 2	13 ± 4
Polyunsaturated (% of E)	8 ± 2	7 ± 2	8 ± 2	8 ± 3
Omega-6/omega-3 ratio	7.3 ± 2.5	7.0 ± 1.9	8.1 ± 2.2	7.7 ± 2.3

Mean ± SD. * different from “household members”, *p* < 0.05 ANOVA and Games-Howell post hoc test. ^1^ mono- and di-saccharides, ^2^ plant cell wall polymeric sugars.

**Table 3:** Intake of selected micronutrients and food groups, and healthy eating index.

	FM (56)	First-Degree Relatives (10)	Household Members (18)	Unrelated Controls (40)
*Vitamins*				
Vitamin C (mg)	114 ± 77	97 ± 62	92 ± 59	132 ± 112
Folate (μg)	371 ± 180	354 ± 110	375 ± 111	424 ± 161
ß-carotene (mg)	4.3 ± 3.5	2.5 ± 1.9	3.0 ± 1.9	4.5 ± 4.9
Vitamin D (μg)	5.6 ± 3.5	4.8 ± 3.3	4.6 ± 4.1	4.5 ± 3.1
Vitamin E (mg)	10 ± 5	8 ± 3	10 ± 4	12 ± 7
Vitamin K (μg)	176 ± 201	121 ± 70	157 ± 127	243 ± 279
*Minerals*				
Calcium (mg)	872 ± 358	926 ± 337	944 ± 458	906 ± 378
Iron (mg)	13 ± 4	12 ± 3	14 ± 4	14 ± 5
Magnesium (mg)	317 ± 94	290 ± 61	334 ± 97	362 ± 138
Potassium (g)	3.0 ± 0.9	2.8 ± 0.7	2.9 ± 0.9	3.2 ± 1.3
Zinc (mg)	11 ± 4	11 ± 4	13 ± 4	11 ± 4
Copper (mg)	1.3 ± 0.6	1.3 ± 0.6	1.3 ± 0.4	1.6 ± 0.6
*Non-nutrients*				
Caffeine (mg)	128 ± 156	116 ± 132	159 ± 130	128 ± 114
*Food Groups* (*servings*)				
Fruits ^1^	1.6 ± 1.1	1.2 ± 0.9	1.1 ± 0.7	2.0 ± 3.1
Vegetables ^1^	1.9 ± 1.0	1.9 ± 0.7	1.8 ± 0.8	2.3 ± 1.3
Grains ^2^	5.0 ± 2.3	6.3 ± 2.1	7.1 ± 2.3	6.1 ± 2.4
Whole grains ^2^	0.9 ± 0.8	0.5 ± 0.5	1.3 ± 1.1	1.2 ± 1.4
Protein foods (animal) ^2^	4.4 ± 3.0	4.4 ± 2.0	4.8 ± 2.8	4.0 ± 2.2
Protein foods (plant) ^2^	1.6 ± 1.5	1.2 ± 1.2	1.6 ± 1.6	2.0 ± 2.2
Dairy ^1^	1.7 ± 1.2	2.2 ± 1.1	2.0 ± 1.4	1.7 ± 1.1
Yogurt ^1^	0.2 ± 0.3	0.2 ± 0.2	0.1 ± 0.2	0.2 ± 0.3
Alcoholic drinks ^3^	0.6 ± 1.4	0.3 ± 0.4	0.5 ± 0.9	0.5 ± 1.1
Diet quality (HEI 2015)	58 ± 16	52 ± 8	53 ± 12	55 ± 15

Vitamin D: ergocalciferol + cholecalciferol; vitamin E: α-tocopherol; vitamin K: phylloquinone. ^1^ Servings in cup equivalents; ^2^ servings in ounce equivalents; ^3^ as number of drinks; HEI: Healthy Eating Index, see Methods for elaboration. A table summarizing the daily average intakes of macronutrients has been previously reported [10].

## Data Availability

All data generated as part of this study are included in this published article and its supplementary file or are available from the corresponding authors on reasonable request. Genome sequences can be found at: https://www.ncbi.nlm.nih.gov/sra/PRJNA521398.

## Supplementary Materials

The following supporting information can be downloaded at: https://www.mdpi.com/article/10.3390/ijerph19063254/s1, Figure S1: Consort diagram of the study.; Figure S2: Non-metric multidimensional scaling (NMDS) analysis; Table S1: Fibromyalgia related variables; Table S2: Nutritional supplements taken by study participants categorized by classes.

## References

[1] Häuser W., Ablin J., Fitzcharles M.-A., Littlejohn G., Luciano J.V., Usui C., Walitt B. (2015). Fibromyalgia. Nat. Rev. Dis. Primers.

[2] Rahman A., Underwood M., Carnes D. (2014). Fibromyalgia. BMJ.

[3] Sluka K.A., Clauw D.J. (2016). Neurobiology of Fibromyalgia and Chronic Widespread Pain. Neuroscience.

[4] Mansfield K.E., Sim J., Jordan J.L., Jordan K.P. (2016). A Systematic Review and Meta-Analysis of the Prevalence of Chronic Widespread Pain in the General Population. Pain.

[5] Clauw D.J. (2014). Fibromyalgia: A Clinical Review. JAMA.

[6] Chinn S., Caldwell W., Gritsenko K. (2016). Fibromyalgia Pathogenesis and Treatment Options Update. Curr. Pain Headache Rep..

[7] Busch A.J., Webber S.C., Brachaniec M., Bidonde J., Bello-Haas V.D., Danyliw A.D., Overend T.J., Richards R.S., Sawant A., Schachter C.L. (2011). Exercise Therapy for Fibromyalgia. Curr. Pain Headache Rep..

[8] Häuser W., Ablin J., Perrot S., Fitzcharles M.-A. (2017). Management of Fibromyalgia: Practical Guides from Recent Evidence-Based Guidelines. Pol. Arch. Intern. Med..

[9] Lowry E., Marley J., McVeigh J.G., McSorley E., Allsopp P., Kerr D. (2020). Dietary Interventions in the Management of Fibromyalgia: A Systematic Review and Best-Evidence Synthesis. Nutrients.

[10] Minerbi A., Gonzalez E., Brereton N.J.B., Anjarkouchian A., Dewar K., Fitzcharles M.-A., Chevalier S., Shir Y. (2019). Altered microbiome composition in individuals with fibromyalgia. Pain.

[11] Clos-Garcia M., Andrés-Marin N., Fernández-Eulate G., Abecia L., Lavín J.L., van Liempd S., Cabrera D., Royo F., Valero A., Errazquin N. (2019). Gut Microbiome and Serum Metabolome Analyses Identify Molecular Biomarkers and Altered Glutamate Metabolism in Fibromyalgia. EBioMedicine.

[12] Human Microbiome Project Consortium (2012). Structure, Function and Diversity of the Healthy Human Microbiome. Nature.

[13] Graf D., Di Cagno R., Fåk F., Flint H.J., Nyman M., Saarela M., Watzl B. (2015). Contribution of Diet to the Composition of the Human Gut Microbiota. Microb. Ecol. Health Dis..

[14] Singh R.K., Chang H.-W., Yan D., Lee K.M., Ucmak D., Wong K., Abrouk M., Farahnik B., Nakamura M., Zhu T.H. (2017). Influence of Diet on the Gut Microbiome and Implications for Human Health. J. Transl. Med..

[15] Murtaza N., Cuív P.Ó., Morrison M. (2017). Diet and the Microbiome. Gastroenterol. Clin. N. Am..

[16] Rajilić-Stojanović M., Jonkers D.M., Salonen A., Hanevik K., Raes J., Jalanka J., de Vos W.M., Manichanh C., Golic N., Enck P. (2015). Intestinal Microbiota and Diet in IBS: Causes, Consequences, or Epiphenomena?. Am. J. Gastroenterol..

[17] Distrutti E., Monaldi L., Ricci P., Fiorucci S. (2016). Gut Microbiota Role in Irritable Bowel Syndrome: New Therapeutic Strategies. World J. Gastroenterol..

[18] Staudacher H.M., Whelan K. (2017). The Low FODMAP Diet: Recent Advances in Understanding Its Mechanisms and Efficacy in IBS. Gut.

[19] Olson C.A., Vuong H.E., Yano J.M., Liang Q.Y., Nusbaum D.J., Hsiao E.Y. (2018). The Gut Microbiota Mediates the Anti-Seizure Effects of the Ketogenic Diet. Cell.

[20] Arranz L.-I., Canela M.-Á., Rafecas M. (2012). Dietary Aspects in Fibromyalgia Patients: Results of a Survey on Food Awareness, Allergies, and Nutritional Supplementation. Rheumatol. Int..

[21] Ruiz-Cabello P., Soriano-Maldonado A., Delgado-Fernandez M., Alvarez-Gallardo I.C., Segura-Jimenez V., Estevez-Lopez F., Camiletti-Moirón D., Aparicio V.A. (2017). Association of Dietary Habits with Psychosocial Outcomes in Women with Fibromyalgia: The al-Ándalus Project. J. Acad. Nutr. Diet..

[22] López-Rodríguez M.M., Granero Molina J., Fernández Medina I.M., Fernández Sola C., Ruiz Muelle A. (2017). Patterns of Food Avoidance and Eating Behavior in Women with Fibromyalgia. Endocrinol. Diabetes Y Nutr..

[23] Wolfe F., Clauw D.J., Fitzcharles M.-A., Goldenberg D.L., Häuser W., Katz R.L., Mease P.J., Russell A.S., Russell I.J., Walitt B. (2016). 2016 Revisions to the 2010/2011 Fibromyalgia Diagnostic Criteria. Semin. Arthritis Rheum..

[24] Burckhardt C.S., Clark S.R., Bennett R.M. (1991). The Fibromyalgia Impact Questionnaire: Development and Validation. J. Rheumatol..

[25] Morin C.M. (1993). Insomnia: Psychological Assessment and Management.

[26] Vol S., Bedouet M., Gusto G., Leglu C., Beslin E., Decou P., Nègre E., Planage B., Chazelle E., Mercier F. (2011). Evaluating Physical Activity: The AQAP Questionnaire and Its Interpretation Software. Ann. Phys. Rehabil. Med..

[27] Fitzcharles M.-A., Ste-Marie P.A., Panopalis P., Ménard H., Shir Y., Wolfe F. (2012). The 2010 American College of Rheumatology Fibromyalgia Survey Diagnostic Criteria and Symptom Severity Scale Is a Valid and Reliable Tool in a French Speaking Fibromyalgia Cohort. BMC Musculoskelet. Disord..

[28] Perrot S., Dumont D., Guillemin F., Pouchot J., Coste J., French Group for Quality of Life Research (2003). Quality of Life in Women with Fibromyalgia Syndrome: Validation of the QIF, the French Version of the Fibromyalgia Impact Questionnaire. J. Rheumatol..

[29] Chahoud M., Chahine R., Salameh P., Sauleau E.A. (2017). Reliability, Factor Analysis and Internal Consistency Calculation of the Insomnia Severity Index (ISI) in French and in English among Lebanese Adolescents. ENeurologicalSci.

[30] Timon C.M., van den Barg R., Blain R.J., Kehoe L., Evans K., Walton J., Flynn A., Gibney E.R. (2016). A Review of the Design and Validation of Web- and Computer-Based 24-h Dietary Recall Tools. Nutr. Res. Rev..

[31] Mifflin M.D., St Jeor S.T., Hill L.A., Scott B.J., Daugherty S.A., Koh Y.O. (1990). A New Predictive Equation for Resting Energy Expenditure in Healthy Individuals. Am. J. Clin. Nutr..

[32] Krebs-Smith S.M., Pannucci T.E., Subar A.F., Kirkpatrick S.I., Lerman J.L., Tooze J.A., Wilson M.M., Reedy J. (2018). Update of the Healthy Eating Index: HEI-2015. J. Acad. Nutr. Diet..

[33] Klindworth A., Pruesse E., Schweer T., Peplies J., Quast C., Horn M., Glöckner F.O. (2013). Evaluation of General 16S Ribosomal RNA Gene PCR Primers for Classical and Next-Generation Sequencing-Based Diversity Studies. Nucleic Acids Res..

[34] Gonzalez E., Pitre F.E., Brereton N.J.B. (2019). ANCHOR: A 16S RRNA Gene Amplicon Pipeline for Microbial Analysis of Multiple Environmental Samples. Environ. Microbiol..

[35] Schloss P.D., Westcott S.L., Ryabin T., Hall J.R., Hartmann M., Hollister E.B., Lesniewski R.A., Oakley B.B., Parks D.H., Robinson C.J. (2009). Introducing Mothur: Open-Source, Platform-Independent, Community-Supported Software for Describing and Comparing Microbial Communities. Appl. Environ. Microbiol..

[36] Love M.I., Huber W., Anders S. (2014). Moderated Estimation of Fold Change and Dispersion for RNA-Seq Data with DESeq2. Genome Biol..

[37] Thorsen J., Brejnrod A., Mortensen M., Rasmussen M.A., Stokholm J., Al-Soud W.A., Sørensen S., Bisgaard H., Waage J. (2016). Large-Scale Benchmarking Reveals False Discoveries and Count Transformation Sensitivity in 16S RRNA Gene Amplicon Data Analysis Methods Used in Microbiome Studies. Microbiome.

[38] Anders S., McCarthy D.J., Chen Y., Okoniewski M., Smyth G.K., Huber W., Robinson M.D. (2013). Count-Based Differential Expression Analysis of RNA Sequencing Data Using R and Bioconductor. Nat. Protoc..

[39] Love M.I., Anders S., Kim V., Huber W. (2015). RNA-Seq Workflow: Gene-Level Exploratory Analysis and Differential Expression. F1000Research.

[40] Beauchesne A.R., Price L.L., Wang C., Chung M. (2020). Dietary Quality is Associated with Better Self-Efficacy and Depression in Patients with Fibromyalgia from a Comparative Effectiveness Trial: A Small Pilot Study. J. Maine Med. Center.

[41] Steinemann N., Grize L., Ziesemer K., Kauf P., Probst-Hensch N., Brombach C. (2017). Relative Validation of a Food Frequency Questionnaire to Estimate Food Intake in an Adult Population. Food Nutr. Res..

[42] Reedy J., Lerman J.L., Krebs-Smith S.M., Kirkpatrick S.I., Pannucci T.E., Wilson M.M., Subar A.F., Kahle L.L., Tooze J.A. (2018). Evaluation of the Healthy Eating Index-2015. J. Acad. Nutr. Diet..

[43] Panizza C.E., Shvetsov Y.B., Harmon B.E., Wilkens L.R., Le Marchand L., Haiman C., Reedy J., Boushey C.J. (2018). Testing the Predictive Validity of the Healthy Eating Index-2015 in the Multiethnic Cohort: Is the Score Associated with a Reduced Risk of All-Cause and Cause-Specific Mortality?. Nutrients.

[44] Holscher H.D., Guetterman H.M., Swanson K.S., An R., Matthan N.R., Lichtenstein A.H., Novotny J.A., Baer D.J. (2018). Walnut Consumption Alters the Gastrointestinal Microbiota, Microbially Derived Secondary Bile Acids, and Health Markers in Healthy Adults: A Randomized Controlled Trial. J. Nutr..

[45] Basson A., Trotter A., Rodriguez-Palacios A., Cominelli F. (2016). Mucosal Interactions between Genetics, Diet, and Microbiome in Inflammatory Bowel Disease. Front. Immunol..

[46] La Rosa S.L., Leth M.L., Michalak L., Hansen M.E., Pudlo N.A., Glowacki R., Pereira G., Workman C.T., Arntzen M.Ø., Pope P.B. (2019). The Human Gut Firmicute Roseburia Intestinalis Is a Primary Degrader of Dietary β-Mannans. Nat. Commun..

[47] Chung W.S.F., Walker A.W., Louis P., Parkhill J., Vermeiren J., Bosscher D., Duncan S.H., Flint H.J. (2016). Modulation of the Human Gut Microbiota by Dietary Fibres Occurs at the Species Level. BMC Biol..

[48] Panche A.N., Diwan A.D., Chandra S.R. (2016). Flavonoids: An Overview. J. Nutr. Sci..

[49] Robert C., Chassard C., Lawson P.A., Bernalier-Donadille A. (2007). *Bacteroides cellulosilyticus* Sp. Nov., a Cellulolytic Bacterium from the Human Gut Microbial Community. Int. J. Syst. Evol. Microbiol..

[50] Pompei A., Cordisco L., Amaretti A., Zanoni S., Matteuzzi D., Rossi M. (2007). Folate Production by Bifidobacteria as a Potential Probiotic Property. Appl. Environ. Microbiol..

[51] Shen S., Lim G., You Z., Ding W., Huang P., Ran C., Doheny J., Caravan P., Tate S., Hu K. (2017). Gut Microbiota Is Critical for the Induction of Chemotherapy-Induced Pain. Nat. Neurosci..

[52] Marum A.P., Moreira C., Saraiva F., Tomas-Carus P., Sousa-Guerreiro C. (2016). A Low Fermentable Oligo-Di-Mono Saccharides and Polyols (FODMAP) Diet Reduced Pain and Improved Daily Life in Fibromyalgia Patients. Scand. J. Pain.

[53] Silva A.R., Bernardo A., Costa J., Cardoso A., Santos P., de Mesquita M.F., Vaz Patto J., Moreira P., Silva M.L., Padrão P. (2019). Dietary Interventions in Fibromyalgia: A Systematic Review. Ann. Med..

[54] Slim M., Calandre E.P., Garcia-Leiva J.M., Rico-Villademoros F., Molina-Barea R., Rodriguez-Lopez C.M., Morillas-Arques P. (2017). The Effects of a Gluten-Free Diet Versus a Hypocaloric Diet Among Patients With Fibromyalgia Experiencing Gluten Sensitivity-like Symptoms: A Pilot, Open-Label Randomized Clinical Trial. J. Clin. Gastroenterol..

[55] Brain K., Burrows T.L., Rollo M.E., Hayes C., Hodson F.J., Collins C.E. (2019). The Effect of a Pilot Dietary Intervention on Pain Outcomes in Patients Attending a Tertiary Pain Service. Nutrients.

[56] Koudoufio M., Desjardins Y., Feldman F., Spahis S., Delvin E., Levy E. (2020). Insight into Polyphenol and Gut Microbiota Crosstalk: Are Their Metabolites the Key to Understand Protective Effects against Metabolic Disorders?. Antioxidants.

[57] Wang J., Chen Y., Hu X., Feng F., Cai L., Chen F. (2020). Assessing the Effects of Ginger Extract on Polyphenol Profiles and the Subsequent Impact on the Fecal Microbiota by Simulating Digestion and Fermentation In Vitro. Nutrients.

