# Supplementary material for: Dietary Intake Is Unlikely to Explain Symptom Severity and Syndrome-Specific Microbiome Alterations in a Cohort of Women with Fibromyalgia
Source: Int J Environ Res Public Health. 2022 Mar 10;19(6):3254. doi: 10.3390/ijerph19063254 (PMC8950034; doi:10.3390/ijerph19063254)
Supplement: Supplementary file 1 [file ijerph-19-03254-s001.zip › ijerph-1521259-supplementary.pdf]

Supplementary figures

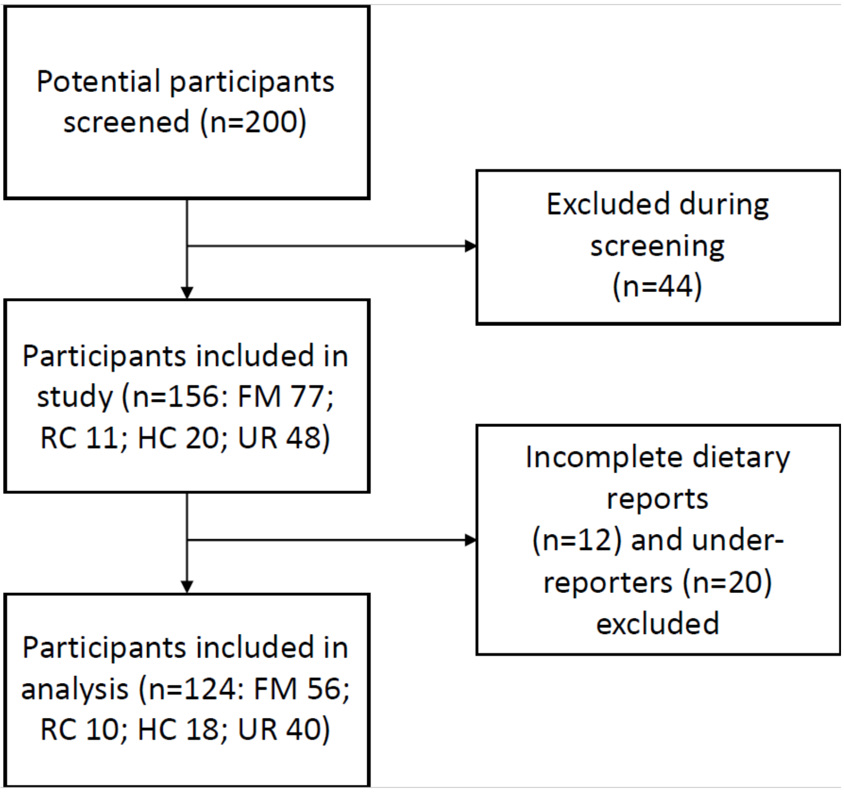

Supplementary Figure S1: Consort diagram of the study.

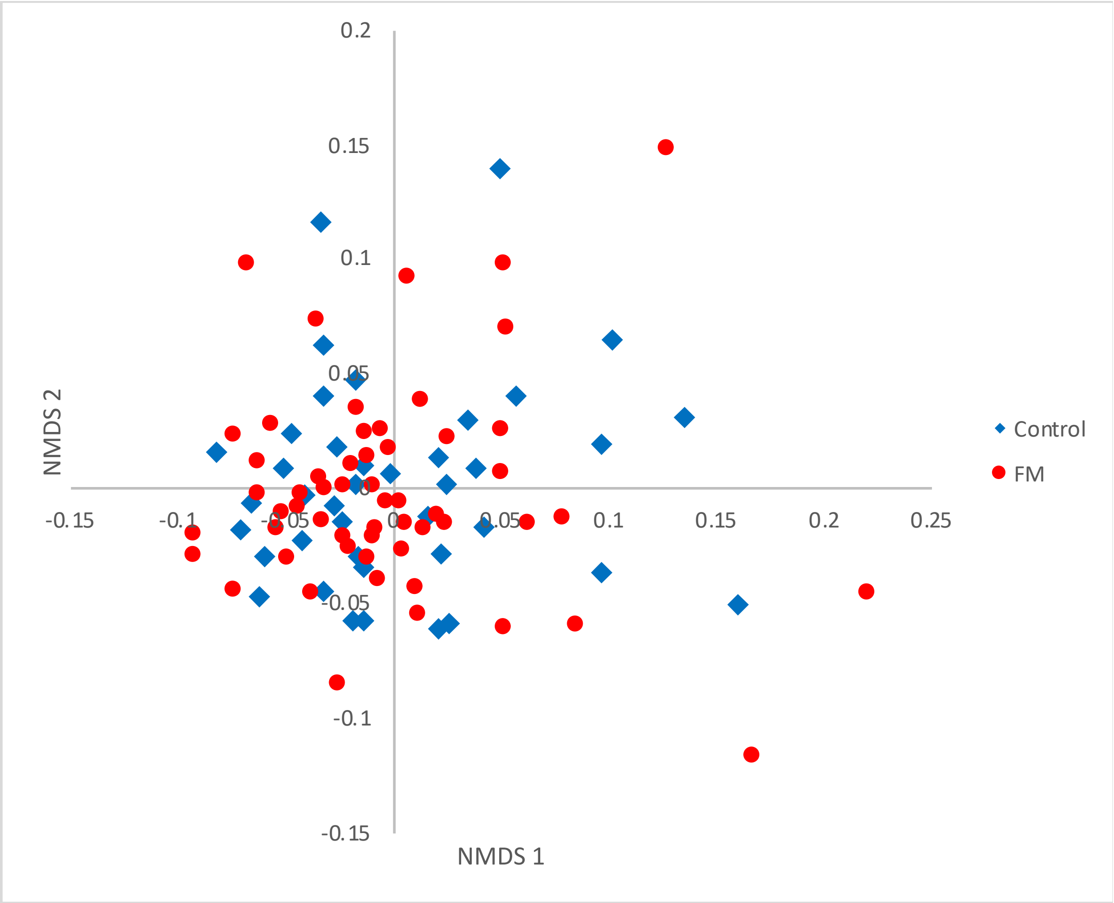

Supplementary figure S2: Non-metric multidimensional scaling (NMDS) analysis. Daily macro- and micronutrient intake among fibromyalgia patients (red circles) vs. all controls (blue diamonds). Control and fibromyalgia factors were projected onto a NMDS diagram and tested by Monte Carlo permutation test (envfit function, R Vegan package). No significant between-group difference was observed (R^2=0.001, p=0.88).

Supplementary Table S1: Fibromyalgia related variables. Years since diagnosis, co-diagnosis of IBS, disease severity and quality of life metrics based on the 2016 fibromyalgia Diagnostic Criteria Widespread Pain Index and Symptom Severity Scale, and on the fibromyalgia Impact Questionnaire (mean±SD or % as indicated). * indicates a significant (p<0.05) difference between fibromyalgia and all control groups; ** indicates a significant (p<0.05) difference between fibromyalgia and HM + UC.

		**FM** (56)	**First degree relatives (10)**	**Household members (18)**	**Unrelated controls (40)**	**ANOVA p**
	Years since diagnosis	11.8±9.7	0	0	0	
	Irritable bowel syndrome	48% *	10%	5.6%	2.5%	<0.0001
FM	Widespread pain index (0-15)	10.3±3.3*	1.8±2.5	2.0±1.3	1.2±1.3	<0.0001
	Fatigue (0-3)	2.6±0.6*	0.9±0.7	1.0±0.7	0.6±0.7	<0.0001
	Waking unrefreshed (0-3)	2.3±0.7*	1.2±0.9	0.9±0.7	0.5±0.8	<0.0001

	Cognitive symptoms (0-3)	2.1±0.8*	0.7±0.9	0.8±07	0.2±0.4	<0.0001
	Headaches	86%**	60%	56%	43%	<0.0001
	Abdominal pain	84%*	30%	33%	20%	<0.0001
	Depression	59%*	30%	17%	18%	<0.0001
	Total symptom severity score (0-12)	9.2±1.8*	4.0±3.1	3.7±1.9	2.2±1.8	<0.0001
Quality of life	Ability to perform tasks (sum, 0-44)	14.5±6.7*	3.9±6.6	4.9±9.6	2.9±4.4	<0.0001
	Days felt well	1.8±1.8*	5.3±2.1	5.9±1.7	6.7±0.6	<0.0001
	Days missed work	0.84±1.6*	0	0	0	0.0001
	Difficulty working (0-10)	4.4±3.2*	0.4±1.3	0.3±0.8	0.0±0.2	<0.0001
	Pain intensity (0-10)	6.7±2.0*	2.4±2.9	1.3±1.6	0.5±0.9	<0.0001
	Felt tired (0-10)	7.5±2.1*	3.3±2.8	2.7±2.1	1.5±1.8	<0.0001
	Unrefreshed sleep (0-10)	7.1±2.3*	2.4±2.5	3.1±2.1	1.3±1.5	<0.0001
	Stiffness (0-10)	7.3±2.0*	2.2±3.0	2.8±2.9	0.5±1.1	<0.0001
	Felt anxious (0-10)	5.6±2.7*	2.3±2.9	3.2±2.7	0.8±1.8	<0.0001
	Felt depressed (0-10)	4.9±2.8*	2.1±2.7	1.3±2.0	0.5±1.2	<0.0001

Supplementary Table S2: Nutritional supplements taken by study participants categorized by classes. Supplements taken by 5% of participants or more are shown. No significant differences were observed following Bonferroni correction for multiple comparisons.

	**FM (56)**	**First degree relatives (10)**	**Household members (18)**	**Unrelated controls (40)**	**ANOVA p**
All nutritional supplements	70%	40%	0%	35%	0.1
Vitamin D	16%	20%	0%	3%	0.04
Calcium	11%	10%	0%	0%	0.09
Curcumin	0%	0%	0%	5%	0.24
Iron	3.6%	0%	0%	12.5%	0.13
Magnesium	8.9%	0%	0%	2.5%	0.27
Vitamin B12	3.6%	10%	0%	0%	0.25
Vitamin C	7.1%	0%	0%	0%	0.17
